# Histological exhibition of the gastroprotective effect of *Moringa oleifera* leaf extract

**DOI:** 10.1007/s00580-017-2594-0

**Published:** 2017-11-14

**Authors:** S. N. Ijioma, E. N. Nwaogazi, A. A. Nwankwo, H. Oshilonya, C. M. Ekeleme, L. U. Oshilonya

**Affiliations:** 1 Department of Veterinary Physiology and Pharmacology, Michael Okpara University of Agriculture, Umudike, Abia Nigeria; 2 Department of Human Physiology, Faculty of Basic Medical and Health Sciences, Abia State University, Uturu, Nigeria; 3 Department of Physical and Health Education, Alvan Ikoku College of Education, Owerri, Imo Nigeria; 4 Department of Medical Laboratory Science, School of Basic Medical Sciences, University of Benin, Benin City, Edo Nigeria; 5 Department of Biochemistry, College of Natural Sciences, Michael Okpara University of Agriculture, Umudike, Abia Nigeria; 6 Department of Biology, College of Education, Agbo, Delta State Nigeria

**Keywords:** Aspirin, Gastroprotection, *Moringa leaf extract*, Mucosa, Rats

## Abstract

The gastroprotective activity of *Moringa oleifera* leaf extract against aspirin-induced ulcers was investigated in rats. Thirty (30) rats under starvation but with access to drinking water for 48 h were divided into 6 groups of 5 animals each. Animals in groups 1 and 2 were pretreated with 0.2 ml normal saline via the oral route. Group 3 received 32 mg/kg cimetidine while those in groups 4, 5 and 6 received oral Moringa leaf extract treatments at doses 200, 400 and 800 mg/kg body weight respectively. Thirty minutes after treatment, all animals in groups 2 to 6 were given 800 mg/kg Aspirin to induce ulcer. Results obtained showed complete erosion of the superficial epithelium with complete loss of the mucus globules and sloughing off of immediate underlying cells and sparsely distributed intraepithelial lymphocytes in the stomach of rats in which no treatment was given and significantly differed from those of the normal control animals which were essentially intact. No significant gastroprotection was observed in rats pretreated with the lowest dose of the extract (200 mg/kg) as a high degree of intestinal mucosal lesions and complete erosion of the surface epithelium with intraepithelial haemorrhage, moderate inflammation and tissue oedema were observed. Pretreatment with 400 mg/kg, however, offered a mild degree of protection with patches of surface epithelial protection and mucus globules, even though there was still predominant disintegration and sloughing off of superficial and underlying epithelial cells. The level of protection was sufficiently increased in animals treated with 800 mg/kg Moringa extract as there was increased protection of surface epithelium with more mucus globules and compared favourably with the effect of Cimetidine in which patches of intact superficial cells were observed. Moringa leaf extract may contain active agents with gastroprotective and mucus enhancing activities and could be harnessed into safe and potent treatment agents for ulcer in addition to providing template for the development of new antiulcer agents.

## Introduction

The gastric mucosa of the normal stomach is under continuous state of physical and chemical attack as it is exposed daily to the potential ravages of acid and pepsin coupled with a wide variety of potentially damaging agents such as certain foods, a range of temperatures, hyperosmolar and abrasive substances, chemical damage from refluxed bile and pancreatic juice, bacterial toxins and damaging drugs. The gastric mucosa is however able to retain its integrity because of the activity of the gastric mucus barrier (Ahmed et al. 2012). The consumption of Aspirin and other nonsteroidal anti-inflammatory drugs (NSAIDs) is today accepted and practiced globally and is established to be a leading cause of the current high prevalence rate of ulcer. With increasing incidences of pain, inflammations and cardiovascular problems, it may be projected that the consumption of aspirin will go even higher. People use aspirin in varying doses for the management of pain and as a blood thinner to prevent the onset of cardiovascular problems like thrombosis. The readily available nature of aspirin coupled with lack of strict regulations regarding its use make the use of aspirin even popular and further increases the incidence of aspirin-induced gastric ulcers. Ulcers occur due to a breakdown of the gastrointestinal tract’s mucosal defence particularly at a site where the mucosal epithelium is exposed to acid and pepsin (Akomas et al. 2014; Hemamalini et al. 2012; Osim 2002). The precipitating factor is therefore the imbalance between the gastric acid secretion and gastric mucosal integrity (Hemamalini et al. 2012). In addition to NSAIDs, other factors which have implicated in the aetiology of ulcers include stress, smoking, nutritional deficiency, prolonged anxiety, emotional stress, haemorrhagic surgical shock, burns, trauma, genetic factors and infection with *Helicobacter pylori* (Hemamalini et al. 2012; Osim 2002; Musumba et al. 2009).

It has been reported that aspirin causes lower gastrointestinal (GIT) injury and bleeding in more than 50% of its users (Byron and Kenneth 2014). It induces ulcers by disrupting the mucus layer of the GIT, thus exposing the tissues to the harmful effects of gastric pepsin and hydrochloric acid and causing diffusion of acid into gastric mucosa which may burst mucosal blood vessels leading to ulcerations and bleeding (Nurhidayah et al. 2014). The main focus in the management/treatment of ulcer is the improvement of mucosal integrity via increase of its quantity and quality and also inhibition/neutralisation of gastric acid secretion in addition to the elimination of *Helicobacter pylori* via the use of suitable antibiotics (Akomas et al. 2014).

Currently, available agents used for ulcer treatment have not yielded desired results and their usefulness have been limited due numerous side effects, hence the need for alternative means of control. For now, plants and plant-based products appear promising in the renewed search for better ulcer treatment. Some medicinal plants reported to have antiulcer properties following studies on experimental animal models include *Musa paradisiaca* (Akomas et al. 2014), *Loranthus micranthus*, *Acalypher wilkisiana* (Ijioma et al. 2015), *Asparagus racemosus*, *Ficus arnottiana*, *Astonia scholaris*, *Azadirachta indica*, *Bauhinia variesata*, *Terminalia chebula* and *Vetiveria ziziinoides* (Gadekar et al. 2010). Although *Moringa oleifera* is reported to possess antiulcer property (Anwar et al. 2007), there appears to be inadequate data to support these claim.

*Moringa oleifera* is a fast growing evergreen deciduous, perennial tree which grows to a height of 10–12 m with trunk which may reach 45 cm. The plant is slender with dropping branches. The leaves are feathery, pale-green, compound tripinnate, 30–60 cm, with many small leaflets. Flowers are white or creamy with fragrant smell and are bisexual while the seeds are dark brown. Almost every part of the plant has food value (James 1983). Studies on *Moringa oleifera* (family *Moringaceae*) has shown that the plant has antioxidant, antimicrobial, anti-inflammatory, antipyretic, antidiabetic, antiulcer, antitumor antidiarrheal and hypocholesteromic properties (Anitha et al. 2011; Anwar et al. 2007; Ghasi et al. 2000). In this current work, the gastric protective and mucus secretion enhancing properties of *Moringa oleifera* leaf extract was investigated and reported.

## Methods

### Collection of plant materials

Fresh leaves of *Moringa oleifera* were collected from a farm settlement in Umuakwela, Obodoahiara in Ahiazu Mbaise Local Government Area of Imo State, Nigeria and were authenticated at the Department of Forestry, College of Natural Resources and Environmental Management, Michael Okpara University of Agriculture, Umudike in the month of November, 2015.

### Preparation of plants extract

The leaf extract was prepared following Soxhlet method described by Jensen (2007). In this method, fresh leaves so collected were dried under shade for 14 days, after which they were pulverised to fine powder using a manual blender. Fifty (50) grams of the powdered sample was introduced into the extraction chamber of the soxhlet extractor and extraction was done using ethanol as solvent. Temperature was maintained at 70 °C throughout the extraction period of 48 h. At the end of the period, the collected extract in ethanol was dried in a laboratory oven at 40 °C to obtain a pasty dark-green extract with a characteristic aromatic smell which weighed 13.60 g and represented 27.20% yield.

### Gastroprotective effect of Moringa extract in rats

Thirty (30) rats under starvation but with access to drinking water for 48 h were divided into 6 groups of 5 animals each. Animals in groups 1 and 2 were pretreated with 0.2 ml normal saline via the oral route. Group 3 received 32 mg/kg cimetidine. Groups 4, 5 and 6 received oral Moringa extract treatments at doses of 200, 400 and 800 mg/kg body weight respectively. Thirty minutes after treatment, all animals in groups 2 to 6 were given 800 mg/kg Aspirin to induce ulcer. Ulcer was not induced in group 1 rats. All administrations were done via the oral route. Two hours after Aspirin administration, all animals were sacrificed by cervical dislocation. The stomach of each animal was carefully isolated, incised along the greater curvature, rinsed in normal saline and transferred into 10% formalin solution for histological study.

### Histological examination of the stomach of rats for gastroprotective activity

Slices of the isolated stomach were fixed in 10% formal saline for 48 h and were processed by placing them in ascending grades of alcohol in the following order, 50% alcohol for 1 h, 70% alcohol for 1 h, first 95% alcohol for 1 h and second 95% alcohol for 1 h 15 min, first absolute alcohol for one and half hours and second absolute alcohol for 2 h to ensure proper dehydration of the tissues. The dehydrated tissues were then transferred to a mixture of equal volumes of alcohol and xylene where they were left overnight and later cleared with two changes of xylene for 1 h each. They were then infiltrated twice for 1 h each with molten paraffin wax in the oven at 60 °C. The tissues were then embedded in paraffin wax, trimmed and mounted on wooden chuck, and then taken to the microtome for sectioning at 5 μm thickness. The sections were floated in floating-out bath from where they were picked with clean albuminized slides. The slides were placed in a staining dish and excess wax was removed by two changes of xylene, hydrated by descending grades of alcohol in the order absolute alcohol, 95% alcohol and 70% alcohol for 2 min each. The slides were taken to water and then stained by infiltrated Ehrlich haematoxylin for 15 min, and then washed in water for 5 min, differentiated in 10% acid alcohol and blued in running tap for 10 min. They were then counter stained with filtered eosine for 2 min. Excess eosine was removed in ascending grades of alcohol in the order 75% alcohol, 95% alcohol and absolute alcohol for 2 min each. They were then cleared in two changes of xylene and each was cover slipped with depex mountant (Clayden 1967; John and Alan 1977). The slides were viewed under a light microscope and selected images were captured using moticam 2.0 digital camera attached to a computer.

## Results

### Histological study of rat’s stomach to show the protective effect of Moringa

**Plate 1:**
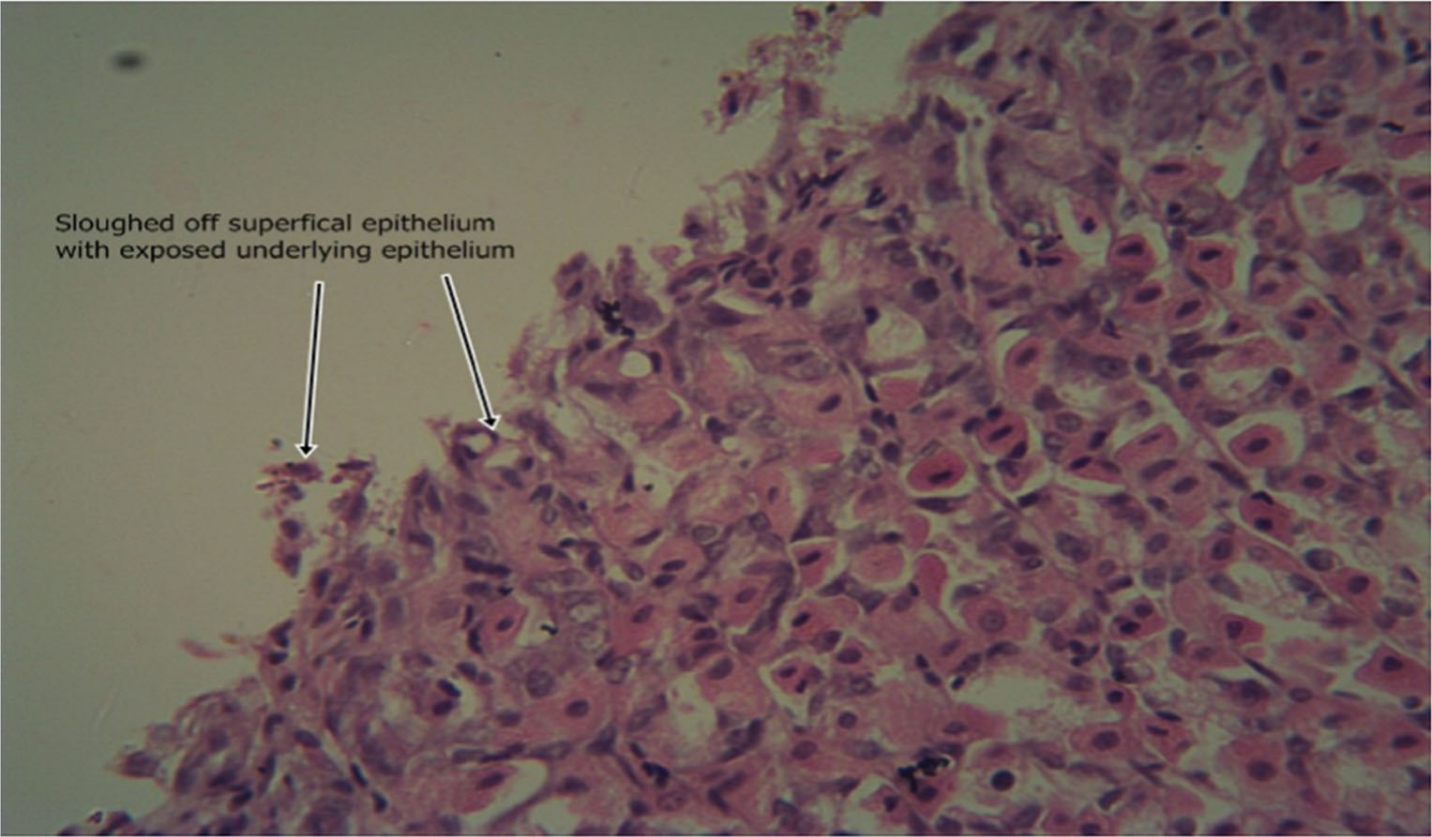
Normal control photomicrograph of stomach showing epithelium with intact superficial cells with protective mucus in small vacuoles (H and E, ×400)

**Plate 2:**
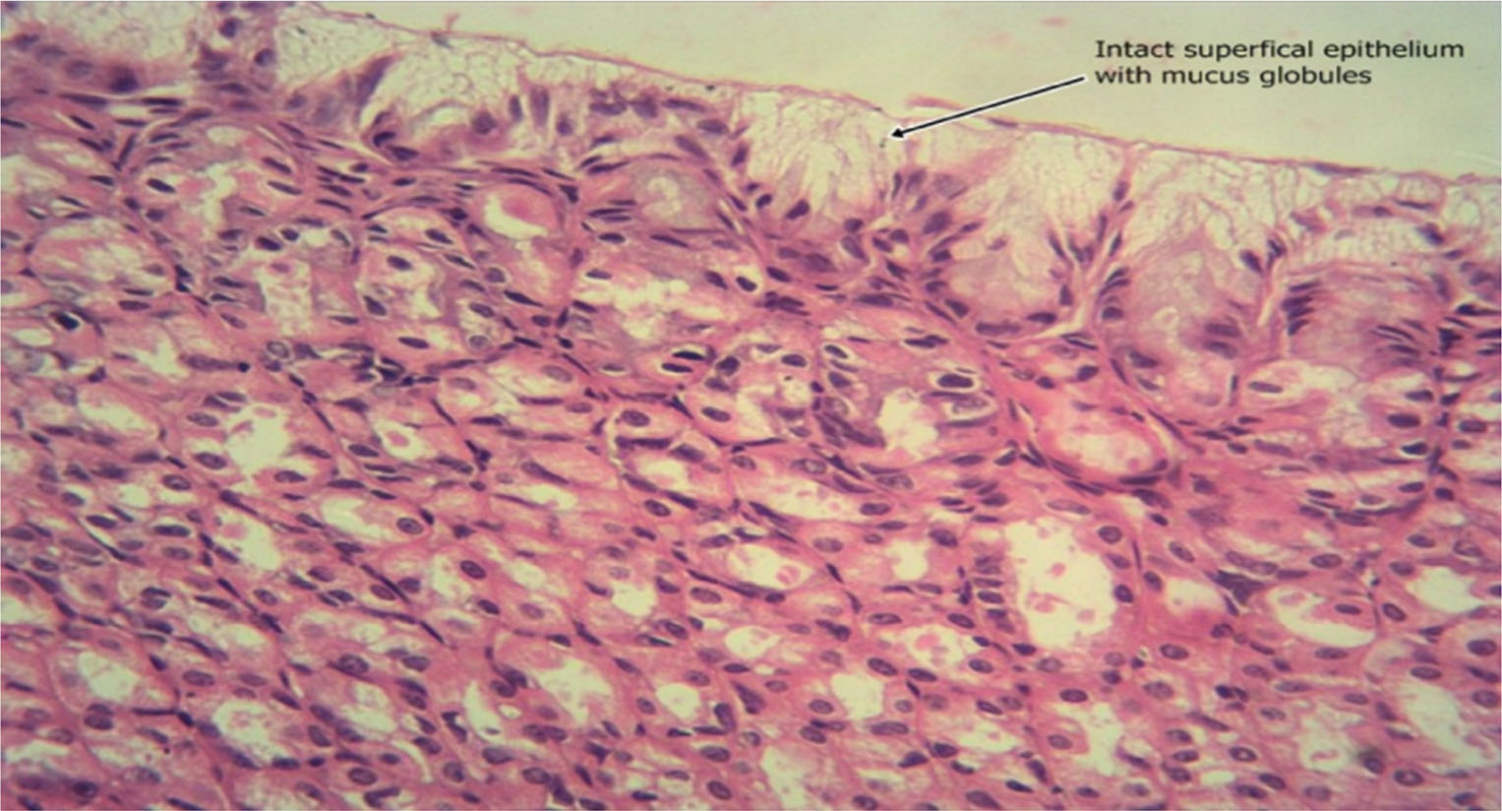
Ulcerated and untreated group’s stomach showing complete erosion of the superficial epithelium with complete loss of the mucus globules and sloughing off of immediate underlying cells. Also seen are sparsely distributed intraepithelial lymphocytes (H and E, ×400)

**Plate 3:**
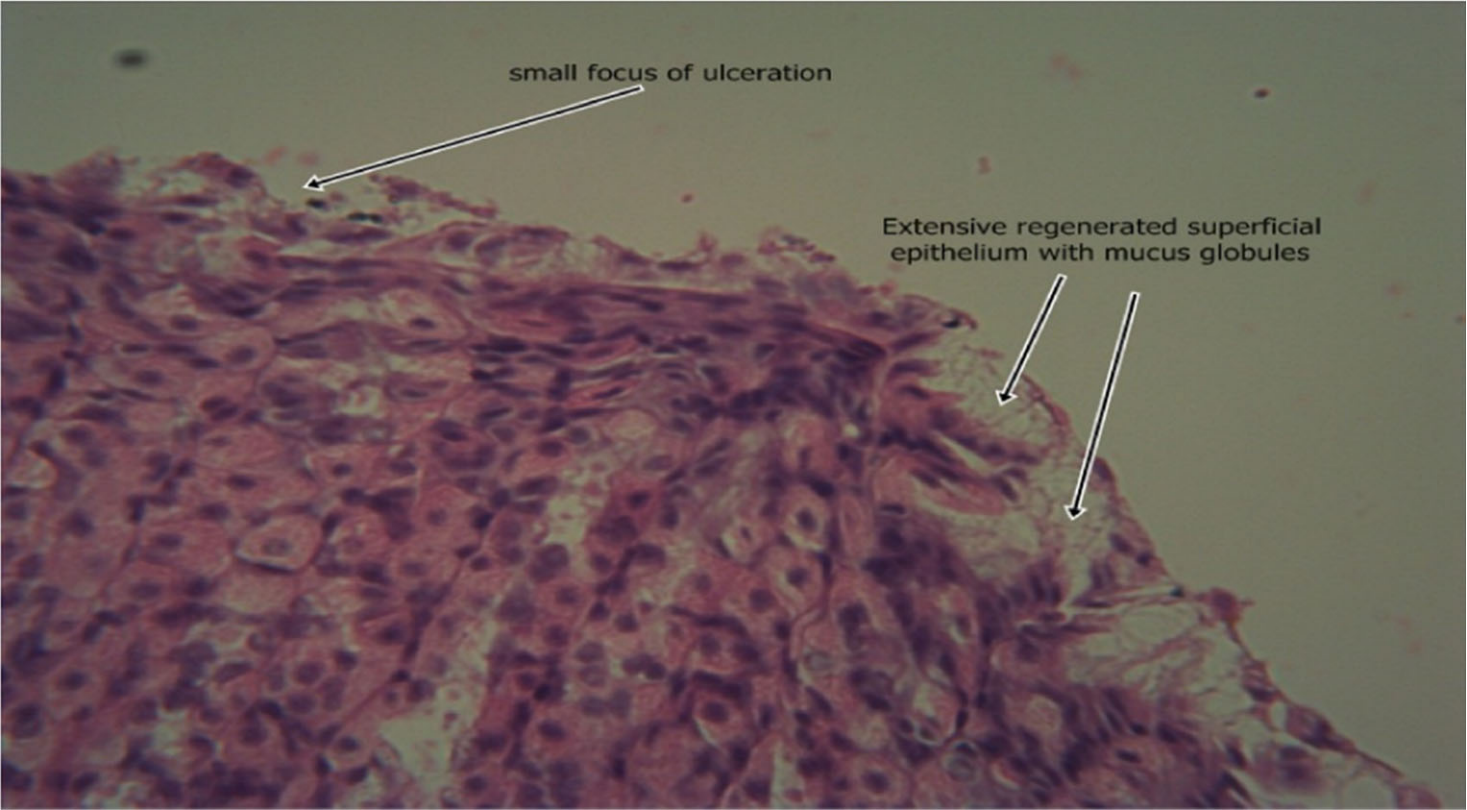
Photomicrograph of Cimetidine-pretreated stomach showing patches of intact superficial cells as well as ulcerated areas with sloughing off of the underlying cell (H and E, ×400)

**Plate 4:**
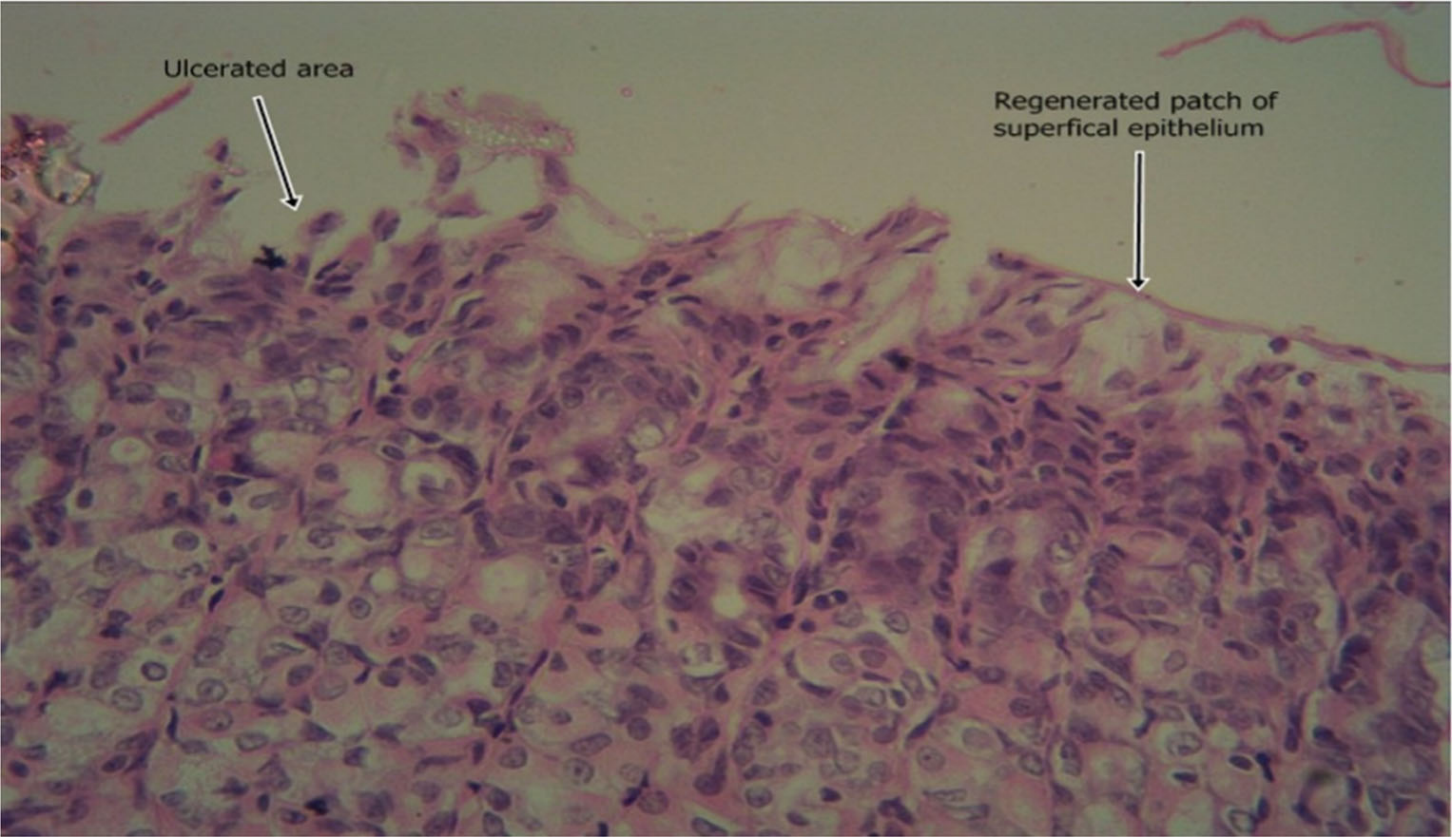
Photomicrograph of stomach which received 200 mg/kg Moringa pretreatment showing complete erosion of the surface epithelium with intraepithelial haemorrhage and moderate inflammation and tissue oedema (H and E, ×400)

**Plate 5:**
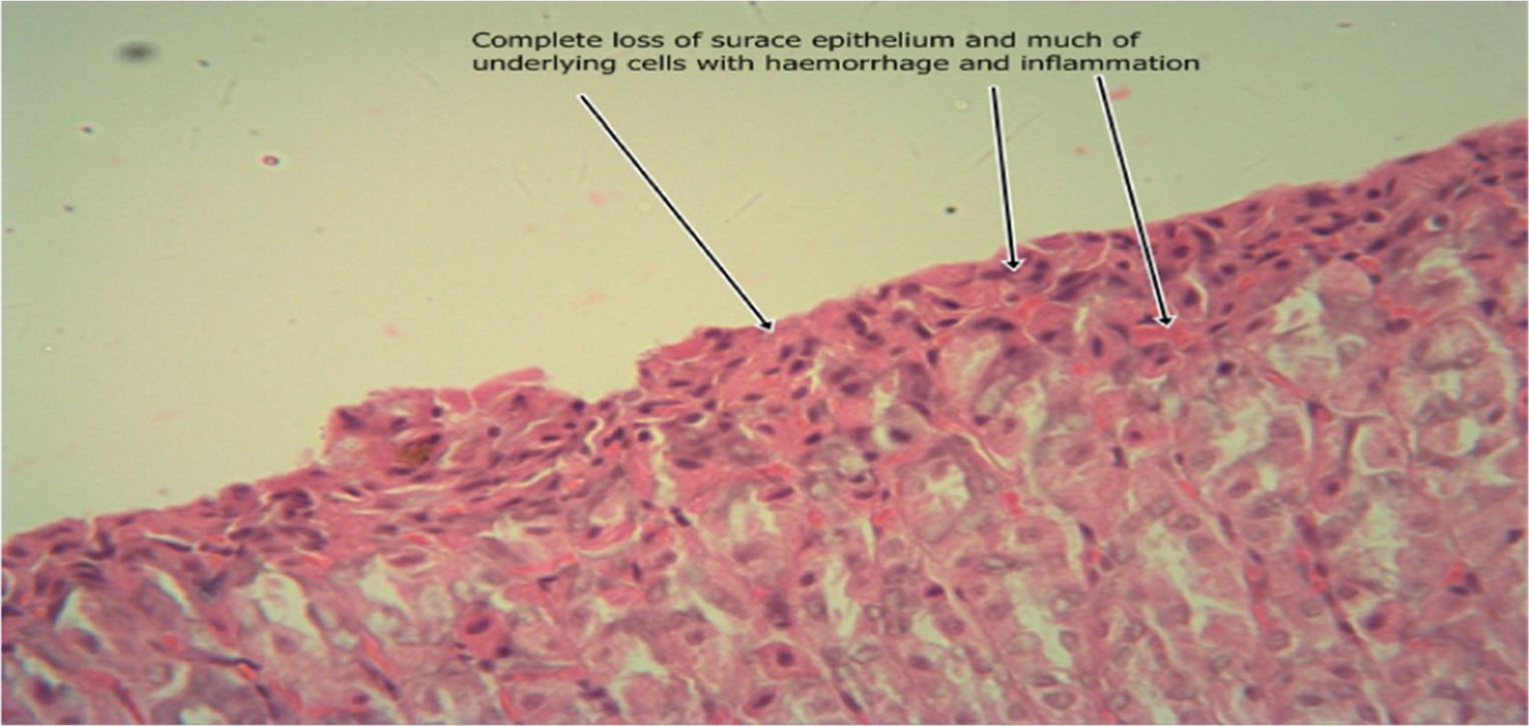
Photomicrograph of stomach 400 mg/kg Moringa pretreatment showing predominant disintegration and sloughing off of superficial and underlying epithelial cells with patches of surface epithelial protection with occasional mucus globules (H and E, ×400)

**Plate 6:**
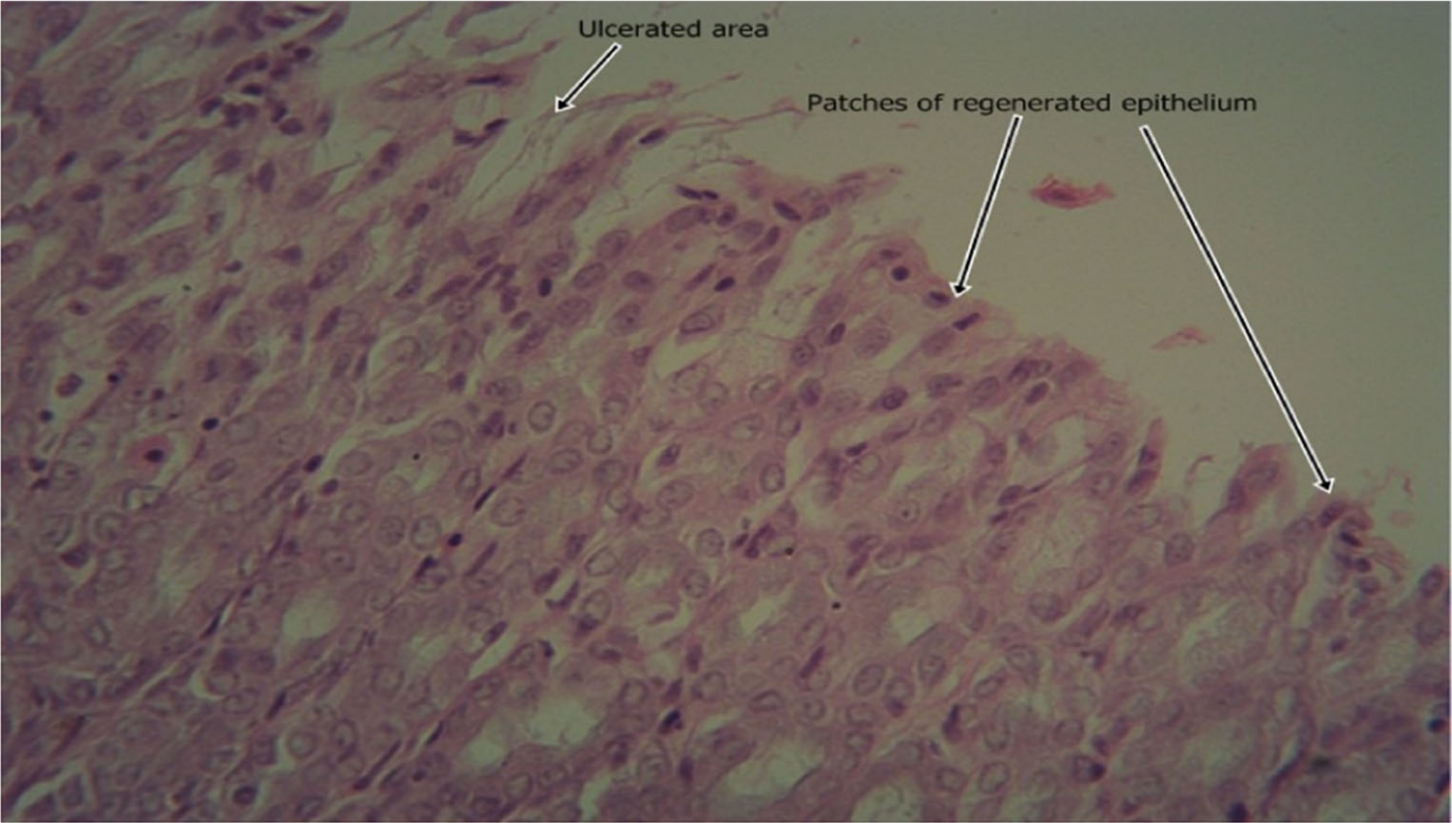
Photomicrograph of stomach pretreated with 800 mg/kg Moringa extract showing increased protection of surface epithelium with more mucus globules than in plate 11 (H and E, ×400)

The results of the intestinal histology of the rat’s stomach designed to investigate the gastroprotective effect of *Moringa oleifera* extract showed that Aspirin (800 mg/kg) could successfully induce gastric ulcerations in rats as the stomach of rats in group 2 treated with this induction agent alone had complete erosion of the superficial epithelium with complete loss of the mucus globules and sloughing off of immediate underlying cells and sparsely distributed intraepithelial lymphocytes (Plate 1). The stomach architecture of group 2 animals were significantly different from those of the normal control animals which were essentially intact (Plate 2). No significant gastroprotection was observed in rats pretreated with the lowest dose of the extract (200 mg/kg) as a high degree of intestinal mucosal lesions and complete erosion of the surface epithelium with intraepithelial haemorrhage, moderate inflammation and tissue oedema were observed (Plate 3), and differ significantly with that of the normal control animals in which the epithelium, superficial cells and protective mucus in small vacuoles were all intact (Plate 2). Pretreatment with 400 mg/kg, however, offered a mild degree of protection with patches of surface epithelial protection and occasional mucus globules, even though there was still predominant disintegration and sloughing off of superficial and underlying epithelial cells (Plate 4). The level of protection was sufficiently increased in animals treated with 800 mg/kg Moringa extract as there was increased protection of surface epithelium with more mucus globules (Plate 5), comparing favourably with the effect of Cimetidine as observed in group 3 in which patches of intact superficial cells were observed (Plate 6).

## Discussion

Results of this work has shown that Moringa extract provided sufficient protection against aspirin-induced gastric ulcers, with evidence of mucus membrane enhancing activity. Aspirin induces gastric ulcerations by reducing the hydrophobicity of the mucus gel layer by changing the action of surface-active phospholipids and suppression of prostaglandin synthesis (Saeed et al. 2006). Prostaglandin is a key player in the protection of gastric mucosal integrity by increasing local blood flow and promoting synthesis and secretion of mucus and bicarbonate (Byron and Kenneth 2014). While the bicarbonate offers protection by lowering the acidity of the gastric lumen, the mucus layer serves as a barrier which protects against the effect of pepsin and also hydrochloric acid which in most cases are the initiators of ulcerations (Nurhidayah et al. 2014). The breakdown of this cascade of body defence activities by a nonsteroidal anti-inflammatory drug (NSAID) like aspirin is responsible for the development of ulcerations characterised by mucosal bleeding (Suleiman et al. 2010; Musumba et al. 2009).

The gastroprotection offered by Moringa extract as evidenced by the histological plates may be due to the presence of phytocomponents such as flavonoids, tannins, terpenoids, sterols, alkaloids and phenols which have been reported to be present in the leaves extract of Moringa. These phytochemical agents have indeed given positive results when tested for antiulcer and gastroprotective properties (Noemi et al. 2014; Gadekar et al. 2010). Lewis et al. (1999) and Kumar et al. (2013) reported the usefulness of flavonoids in wound healing promotion, cellular regeneration and cytoprotection as key antiulcer dynamics. In view of the fact that ulcer is greatly linked with oxidative stress (Shokouhsadat et al. 2015; Akomas et al. 2014), the antioxidant effects of flavonoids and phenols in Moringa leaf extract may have contributed to the observed antiulcer effect. Separate studies on isolated flavonoids also revealed high levels of gastroprotection in association with the administration of flavonoids (Zayachikiwaka et al. 2005). Phenolic compounds were on the other hand reported to exhibit gastroprotective effects via various mechanisms including antisecretory activity, cytoprotection, modulation of inflammatory mediators, antioxidative stress defence and enhancement of the levels of antioxidant enzymes in the body (Shokouhsadat et al. 2015).

The reported antibacterial activity of Moringa leaf extract may suggest another mechanism of antiulcer effects but leaves room for further evaluation of this antibacterial effect in relation to inhibiting the growth of *Helicobacter pylori* (a major cause of ulcer).

## Conclusion

Moringa leaf extract may contain active agents with gastroprotective and mucus-enhancing activities and could be harnessed into safe and potent treatment agents for ulcers and may provide template for the development of new antiulcer agents.
